# Subsequent Malignant Neoplasm of Bone in Children and Adolescent—Possibility of Multimodal Treatment

**DOI:** 10.3390/curroncol29020085

**Published:** 2022-02-11

**Authors:** Anna Raciborska, Katarzyna Bilska, Tomasz Koziński, Carlos Rodriguez-Galindo

**Affiliations:** 1 Department of Oncology and Surgical Oncology for Children and Youth, Institute of Mother and Child, 01 -211 Warsaw, Poland; katarzyna.bilska@imid.med.pl (K.B.); tomasz.kozinski@imid.med.pl (T.K.); 2 Departments of Global Pediatric Medicine and Oncology, St. Jude Children’s Research Hospital, Memphis, TN 38105, USA; carlos.rodriguez-galindo@stjude.org

**Keywords:** subsequent malignant neoplasm, multimodal therapy, children, adolescents, bone tumors

## Abstract

Background: In recent years, modifications of treatment protocols introduced in pediatric oncology have resulted in a significant improvement in treatment outcomes. Unfortunately, the probability of subsequent malignant neoplasm (SMN) in this group of patients is 3 to 6 times higher than the general age-matched population. In this study, we sought to evaluate the treatment options for patients with secondary bone tumors after prior anti-cancer therapy. Materials and Methods: Twenty-four patients (median age 12.9 years) with subsequent malignant bone tumors were treated according to oncological guidelines for bone sarcoma during the period 1991–2020. All patients had a standard tumor imaging and laboratory evaluation. All toxicities were documented. Results: The median time from the first neoplasm to SMN was 7.6 years (range 2.4 to 16.3 years). All patients received chemotherapy and underwent surgery as a local control procedure. Two patients with Ewing sarcoma had additional radiation on the tumor bed. A complete response was achieved in 20 patients. With a median follow-up of 18.3 years (range 5.7 to 40.3 years), 18 patients (75%) are alive. The estimated 5-year post-subsequent bone malignant neoplasm survival was 74.5% (95% CI 55–95%). Fourteen patients required chemotherapy dose modification, and doxorubicin was discontinued in seven patients. One patient required a renal transplant two years after treatment. There were no other significant toxicities. Conclusions: The treatment of bone SMNs can be effective, although in many patients it is necessary to reduce the doses of drugs. Early detection and aggressive treatment can improve the outcome.

## 1. Introduction

Improvements in multidisciplinary care for childhood cancer have resulted in a significant improvement in treatment outcomes. While in the 1960s the chance of 5-year overall survival for a child diagnosed with cancer was 20–30%, it is now estimated to be 80% [1]. These improvements translate directly to a growing population of childhood cancer survivors; however, the late effects of cancer treatment are reported in about two-thirds of patients, and in one-fourth of them they are health- or life-threatening [2,3]. A subsequent malignant neoplasm (SMN) belongs to this category, although their occurrence may depend not only on the type of therapy used but also on individual predispositions [4,5,6,7,8,9,10,11,12].

In the studies published so far, the risk of SMN is estimated at 2 to 12% and is related to the type of treatment used, the patient’s age during therapy, sex, and time since the end of the treatment [4,5,6,7,8,9,10,11,12]. Moreover, the probability of a secondary neoplasm increases significantly if genetic predisposition factors to the development of cancers are present [13]. As patients mature and age, acquired or environmental risk factors, such as smoking and excessive sun exposure, also increase the possibility of developing SMN [6,14]. All in all, compared to the population risk, the probability of SMN in the group of patients treated in childhood with cancer increases 3–6 times [4]. Considering the increasing number of survivors, new challenges arise to provide comprehensive anticancer treatment for those who develop an SMN.

In this study, we perform an analysis of treatment options and outcomes for a cohort of patients with secondary bone tumors after prior anti-cancer treatment.

## 2. Materials and Methods

### 2.1. Patients

This retrospective study includes 24 patients with SMN of bone treated with multimodal therapy during the period 1991–2020. During both the first and second treatments, all patients had standard diagnostic procedures, as clinically indicated and available, and all had a histological confirmation of diagnosis. Informed consent was obtained from all patients or their guardians before treatment. Approval for this retrospective study was obtained from all the relevant institutions in compliance with international regulations for protection of human research subjects (Ethics Committee at the Mother and Child Institute; approval code 6/2021; approval date 28 January 2021).

### 2.2. Treatment

#### 2.2.1. First Malignant Neoplasm

In all patients, treatment was conducted according to the existing disease-specific protocols and treatment guidelines, and included different combinations of surgery, chemotherapy, and radiation therapy.

#### 2.2.2. Subsequent Malignant Neoplasm

The following chemotherapy guidelines were the mainstay of treatment: for Ewing sarcoma (ES) patients—the Euro-Ewing regimen (vincristine, ifosfamide, doxorubicin and etoposide as neoadjuvant, and vincristine, actinomycin and cyclophosphamide or ifosfamide as adjuvant); for osteosarcoma patients—European Osteosarcoma Intergroup protocol (doxorubicin, cisplatin and double methotrexate); for chondrosarcoma patients—European Organization for Research and Treatment of Cancer (EORTC) regimen (doxorubicin, cisplatin); and for undifferentiated pleomorphic sarcoma (UPS) and pleomorphic sarcoma patients—PACE regimen (doxorubicin, cisplatin, etoposide, cyclophosphamide). In case of Common Terminology Criteria for Adverse Events (CTCAE) grade 3 toxicity, the doses of cytostatic drugs were reduced (up to 75% of prescribed dose). Doxorubicin was discontinued when the cumulative dose was reached (450 mg/m^2^).

All patients underwent surgery following neoadjuvant chemotherapy, and continued to receive adjuvant chemotherapy after local control, as indicated per protocol. For ES patients with axial tumor, incomplete tumor resection, poor response to chemotherapy (<90% necrosis), or tumor volume > 200 mL, radiation was recommended (45.0–54.0 Gy).

### 2.3. Assessment of Response and Toxicity

All patients had standard tumor imaging using computed tomography (CT), magnetic resonance imaging (MRI), bone scan or positron emission tomography (PET), as indicated and available, prior to starting treatment and every two to three courses. Electrocardiography and echocardiography were performed for every two courses of treatment. Physical examination and laboratory evaluation were performed prior to each cycle or weekly when necessary. All toxicities were documented from day 1 of the first cycle until end of therapy.

The WHO criteria were used to evaluate response; best response was measured according to standard imaging procedures. Thus, a complete response (CR) was defined as no signs of disease. A partial response (PR) was defined as at least a 50% decrease in all measurable lesions (primary or metastases). Progressive disease (PD) was defined as at least a 20% increase in the size of any lesions, or development of new lesions. A stable disease (SD) was defined as absence of CR, PR or PD.

### 2.4. Statistical Methods

Overall survival (OS) was defined as the time interval from the date of diagnosis to the date of death or last follow-up. Time to subsequent malignant neoplasm (TTS) was defined as the time interval from date of diagnosis of the first neoplasm to date of diagnosis SMN. Post-subsequent malignant neoplasm OS was defined as the time interval from date of SMN diagnosis to the date of death or to last follow-up date. Results distributions were estimated using the method of Kaplan-Meier. *p* ≤ 0.05 was regarded as significant. Statistical analysis was performed using STATA 13.3 for Windows.

## 3. Results

Patient characteristics, treatment, and outcome are shown in Table 1 and Table 2.

### 3.1. First Diagnosis and Treatment

The median age at the time of the first diagnosis was 4.9 years (range 0.1 to 15.4 years). Three patients suffered from acute lymphoblastic leukemia, five had Ewing sarcoma, five had soft tissue sarcoma, three had germ cell tumors, four had retinoblastomas, two had Wilms tumor, one had a central nervus system tumor, and one had a neuroblastoma. All patients received chemotherapy and underwent surgery as a local control procedure. Radiation therapy was given in thirteen patients. Two patients received consolidation with high-dose chemotherapy and an autologous (one patient) or allogenic (one patient) hematopoietic stem cell transplant.

### 3.2. Subsequent Malignant Bone Neoplasm Diagnosis and Treatment

The median age at the time of SMN was 12.9 years (range 5.9 to 23.4 years). The median time from the first neoplasm to SMN was 7.6 years (range 2.4 to 16.3 years). Seven patients had metastatic disease at diagnosis of SMN. Thirteen patients had osteosarcoma, three patients had Ewing sarcoma, and five had chondrosarcomas. Two patients were diagnosed with undifferentiated pleomorphic sarcoma (UPS) and one with pleomorphic sarcoma. SMN developed in the irradiated site in 11 out of the 13 patients who received radiation during the treatment of the first malignancy (five patients with ES, one with a brain tumor, three with soft tissue sarcomas, and two with Wilms tumors). Three of four patients with retinoblastoma had positive family cancer history and a confirmed germline *RB1* mutation. All patients received chemotherapy and underwent surgery as a local control procedure. Two patients with ES had additional radiation (45.0 Gy) on the tumor bed.

### 3.3. Toxicity

Fourteen patients required treatment modifications: five patients due to renal disfunction, one of which required renal transplant two years after the end of the SMN treatment (as a result of increasing renal failure); and nine patients due to blood adverse events (grade 3 or 4 thrombocytopenia and/or grade 3 anaemia and/or grade 4 neutropenia according the CTCAE; for this reason they received platelet transfusion and/or red cell transfusion and/or hematopoietic growth factor support). The discontinuation of doxorubicin during the SMN treatment after reaching the maximum cumulative dose occurred in seven patients, four of them with a history of Ewing sarcoma as a first malignant neoplasm. Cardiac disfunction was not observed. There were no other significant toxicities. Toxicities are depicted in Table 2.

### 3.4. Follow-Up and Outcome

**Figure 1:**
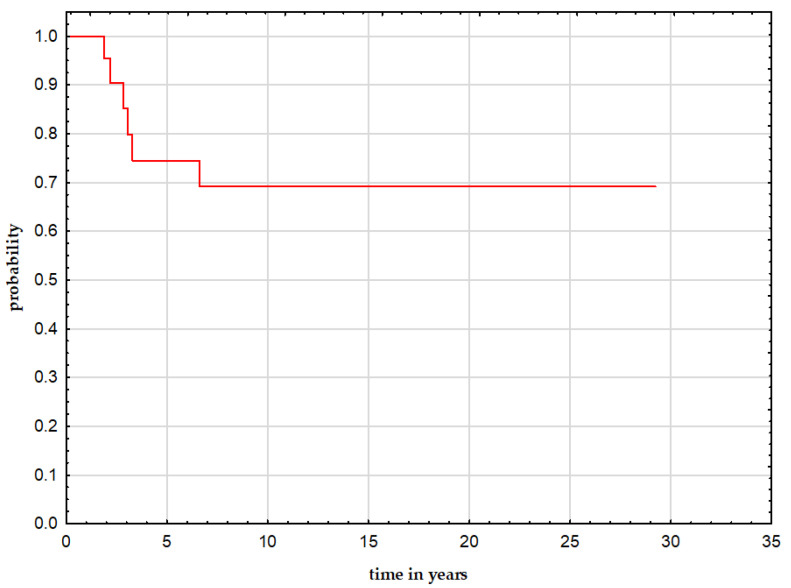
5-year overall survival after SMNs for the whole group.

A CR was achieved in twenty patients (83.3%). With a median follow-up of 18.3 years (range 5.7 to 40.3 years), 18 patients (75%) are alive. Four patients (16.7%) progressed during therapy, and they all died; one of them received radiation on a tumor bed during the first treatment, and two of them had a germline *RB1* mutation. Two patients died due to a third malignant neoplasm (Table 2); one of them received radiation on a tumor bed during the first treatment, and the other had a germline *RB1* mutation. In one patient, the mutation result is unknown. The estimated 5-year post-subsequent bone malignant neoplasm was 74.5% (95% CI 55–95%) (Figure 1). Patients with localized disease had a better outcome than patients with metastatic disease (HR 15.96, 95% CI 1.82–139.93, *p* = 0.0016). It seems that patients with osteosarcoma had worse outcomes than patients with other diseases, but a statistical significance was not proven (HR 0.44 95% CI 0.15–1.28, *p* = 0.09).

## 4. Discussion

The number of SMNs is increasing annually, and they now constitute one in six of all reported neoplasms [14]. In a systematic review study conducted by Caruso, the cumulative incidence rates of SMN ranged from 0.9 to 8.4% and 10.1 to 20.5% at 5 and 30 years after the initial diagnosis [15]. Two-thirds of the reported SMNs were solid tumors, although acute myeloid leukemia/myelodysplastic syndrome was the single most-diagnosed SMN, with generally poor outcomes. Osteosarcoma is one of the most common SMNs of the bone, followed by Ewing sarcoma and chondrosarcoma, which is very rare [16]. In our study, osteosarcoma occurred in 13 of 24 patients, chondrosarcoma in five cases, and Ewing sarcoma in three cases.

SMN can be divided into three main groups: those arising as a consequence of anti-cancer treatment (70% appear within the radiation field [13]), those resulting from genetic susceptibility, and those resulting from exposure to environmental factors (e.g., smoking) [14]. However, many cases are multifactorial and it may be difficult to determine the cause of the SMN. In our group, 13 patients had received prior radiotherapy and genetic susceptibility was documented in three cases.

With the increase in the number of childhood cancer survivors that developed an SMN, strategies for their multidisciplinary management need to be considered. The treatment for the subsequent cancer depends on the type of therapy used in the first treatment, and thus proper tailoring and adaptation is required. When planning treatment, it is necessary to consider the total doses of drugs, the dose and field of the previous radiotherapy, as well as the organ function.

In our study, we sought to evaluate the treatment options for patients with subsequent malignant bone tumors after prior anti-cancer therapy. Our results showed that the successful treatment of bone SMNs was possible. Although the outcome is satisfactory, it is important to mention that 14 of 24 patients required a reduced dose of the drugs, and 7 patients required a discontinuation of anthracyclines. Importantly, all patients underwent surgical treatment.

While good outcomes for patients with second malignant bone tumors can be reached with intensive multidisciplinary care, the development of strategies to reduce risk during the management of the primary malignancy is critical.

In summary, our study confirms the possibility for the effective treatment of patients with subsequent malignant bone tumors, although in many cases it is necessary to reduce the doses of drugs. Early detection and aggressive procedures can improve the outcome.

## Figures and Tables

**Table 1:** Patients’ characteristics-FMN.

Pts no.	GD	Age (y) FMN	Type of Disease	Treatment
1	M	3.3	ALL	CHT
2	F	5.5	ALL	CHT, RT, alHSCT
3	M	5.6	ALL	CHT, RT
4	F	7.4	ES	CHT, S, RT
5	F	15.4	ES	CHT, S, RT
6	M	5.2	ES	CHT, S, RT
7	F	12.1	ES	CHT, S, RT
8	F	7.9	ES	CHT, S, RT
9	F	7.3	FA	CHT, S, RT
10	F	3.0	GCT	CHT, S
11	M	0.1	GCT	CHT, S, aHSCT
12	M	3.3	GCT	CHT, S
13	F	7.5	CNS tu	CHT, S, RT
14	M	10.2	MM	CHT, S
15	M	1.6	NBL	CHT, S
16	M	0.5	RBL	CHT, S
17	F	0.2	RBL	CHT, S
18	F	0.1	RBL	CHT, S
19	F	3.2	RBL	CHT, S
20	M	1.9	RMS	CHT, S, RT
21	M	6.0	RMS	CHT, S, RT
22	F	4.7	RMS	CHT, S
23	M	1.6	Wilms	CHT, S, RT
24	M	7.8	Wilms	CHT, S, RT

No, number; GD, gender; M, male; F, female; FMN, first malignant neoplasm; ALL, acute lymphoblastic leukemia; AML, acute myeloid leukemia; GCT, germ cell tumor; CNS, central nervus system tumor; RBL, retinoblastoma; MM, mesenchymoma malignum; FA, fibromatosis aggressive; ES, Ewing sarcoma; NBL, neuroblastoma; y, years; CHT, chemotherapy; S, surgery; RT, radiation therapy; aHSCT, autologous hematopoietic stem cell transplant; alHSCT, allogenic hematopoietic stem cell transplant.

**Table 2:** Patients’ characteristics-SMN.

Pts no.	Age (y) SMN	Type of Disease	Site of SMN	Meta of SMN	Treatment	CHT Modif.	Toxicity (Grade 3–4)	ADM Discont.	Best Resp.	TMN	Outcome Follow-Up (y) from dgn of SMN
1	15.0	Osteosa	Lower limb	no	CHT, S	yes	blood, renal	no	CR	na	AWD (13.9)
2	11.6	ES	Axial	no	CHT, S, RT	yes	blood	no	CR	na	AWD (1.9)
3	14.0	ES	Axial	no	CHT, S, RT	yes	blood	yes	CR	na	AWD (10.8)
4	12.5	UPS	Lower limb	no	CHT, S	yes	blood	yes	CR	na	AWD (11.0)
5	20.3	CHS	Axial	no	CHT, S	yes	blood	yes	CR	na	AWD (23.6)
6	15.2	CHS	Lower limb	yes	CHT, S	yes	blood	yes	CR	na	AWD (17.6)
7	16.1	Osteosa	Lower limb	yes	CHT, S	yes	blood	yes	CR	Pleom.sa	DOD (2.8)
8	11.8	Pleom.sa	Lower limb	no	CHT, S	yes	blood	no	CR	na	AWD (1.9)
9	15.6	CHS	Axial	no	CHT, S	yes	blood	no	CR	na	AWD (17.0)
10	16.1	CHS	Lower limb	no	CHT, S	yes	renal	yes	CR	AML	DOD (6.6)
11	14.6	Osteosa	Lower limb	no	CHT, S	yes	renal	no	CR	na	AWD (0.7)
12	13.2	Osteosa	Axial	no	CHT, S	no	na	no	CR	na	AWD (8.4)
13	9.9	Osteosa	Axial	yes	CHT, S	no	na	no	PD	na	DOD (3.3)
14	20.3	Osteosa	Axial	no	CHT, S	no	na	no	CR	na	AWD (12.2)
15	5.9	Osteosa	Upper limb	no	CHT, S	no	na	no	CR	na	AWD (16.2)
16	5.9	Osteosa	Lower limb	yes	CHT, S	no	na	no	PD	na	DOD (2.2)
17	11.6	Osteosa	Lower limb	yes	CHT, S	no	na	no	PD	na	DOD (3.1)
18	11.2	Osteosa	Lower limb	no	CHT, S	no	na	no	CR	Thyroid tu	AWD (29.2)
19	6.4	ES	Lower limb	no	CHT, S	no	na	no	CR	na	AWD (15.0)
20	8.8	Osteosa	Lower limb	no	CHT, S	yes	renal	no	CR	na	AWD (0.6)
21	12.0	Osteosa	Axial	yes	CHT, S	yes	blood, renal	no	PD	na	DOD (1.9)
22	9.1	CHS	Lower limb	no	CHT, S	no	na	no	CR	na	AWD (2.5)
23	17.9	Osteosa	Axial	yes	CHT, S	yes	blood	yes	CR	na	AWD (16.2)
24	23.4	Pleom.sa	Axial	no	CHT, S	no	na	no	CR	na	AWD (11.9)

No, number; SMN, subsequent malignant neoplasm; TMN, third malignant neoplasm; ES, Ewing sarcoma; CHS, chondrosarcoma; UPS, undifferentiated pleomorphic sarcoma; y, years; CHT, chemotherapy; S, surgery; RT, radiation therapy; ADM, doxorubicin; AWD, alive without disease; DOD dead of disease; dgn, diagnosis.

## Data Availability

Data and material are available upon request.
